# Salivary miRNA panel to detect HPV-positive and HPV-negative head and neck cancer patients

**DOI:** 10.18632/oncotarget.21725

**Published:** 2017-10-10

**Authors:** Yunxia Wan, Dimitrios Vagenas, Carolina Salazar, Liz Kenny, Chris Perry, Diego Calvopiña, Chamindie Punyadeera

**Affiliations:** ^1^ School of Biomedical Sciences, Institute of Health and Biomedical Innovation, Queensland University of Technology (QUT), Kelvin Grove, Brisbane, Australia; ^2^ Institute of Health and Biomedical Innovation, Queensland University of Technology (QUT), Kelvin Grove, Brisbane, Woolloongabba, Queensland, Australia; ^3^ The School of Chemistry and Molecular Biosciences, The University of Queensland, Queensland, Australia; ^4^ The University of Queensland Diamantina Institute, The University of Queensland, The Translational Research Institute, Woolloongabba, Queensland, Australia; ^5^ The School of Medicine, University of Queensland, Queensland, Australia; ^6^ Royal Brisbane and Women’s Hospital, Brisbane, Central Integrated Regional Cancer Service, Queensland Health, Woolloongabba, Queensland, Australia; ^7^ Princess Alexandra Hospital, Woolloongabba, Brisbane, Australia

**Keywords:** head and neck squamous cell carcinomas, human papilloma virus, miRNAs, saliva

## Abstract

Head and neck squamous cell carcinomas (HNSCC) are a heterogeneous group of tumours that originate predominantly from the oral cavity, pharynx and larynx. Our aim was to determine whether salivary miRNA expression levels can diagnose these cancer subtypes. Saliva samples were collected from healthy controls (n=113, smoker and non-smokers), HPV-positive (n=54) and HPV-negative (n=47) HNSCC patients. The miRNA expression levels in saliva was quantified using qPCR. The potential of salivary miRNAs to discriminate these groups of patients was evaluated using multiple logistic regression with ROC analysis and a 10-fold cross-validation analysis. Salivary miRNA-9, -127, -134, -191, -222 and -455 were shown to discriminate a control group from a HPV-negative HNSCC patient group with a sensitivity of 60% and a specificity of 94%; whilst salivary miRNA-9,-134, -196b, -210, and -455 were the most parsimonious subset discriminating a control group from a HPV-positive HNSCC group, with a sensitivity of 65% and a specificity of 95%. Furthermore, miRNA-9, -134, -196b, -210 and -455 as a panel, was the most parsimonious subset to discriminate HPV-positive HNSCC patients from HPV-negative HNSCC patients. In addition, the expression levels of miRNA-9, -127, -196a, -196b, -210, -222 and -455 were significantly increased in the saliva collected from early stage HNSCC patients compared to controls. A future multi-centre confirmatory study is warranted to test the diagnostic performance of these salivary miRNA prior to clinical implementation.

## INTRODUCTION

Head and neck cancers are a heterogeneous group of tumours that originate in the the nasal cavity, paranasal sinuses, oral cavity, salivary glands, pharynx and larynx [1–4]. A large number (>90%) of these malignancies are squamous cell carcinoma (SCC) that arise from the squamous epithelium of the oral cavity and the oropharynx [2, 5–7], they are generally referred to as head and neck squamous cell carcinomas (HNSCC). HNSCC currently stands as the sixth most common cancer worldwide with 900,000 new cases per year, with about 300,000 deaths annually [1].

There is a rapid rise in the incidence of oropharyngeal cancers (OPC), a type of HNSCC, caused by the human papillomavirus type 16 (HPV-16) [8] in the USA and in the western world, whilst oral cavity cancer (OC, predominantly HPV-negative) prevalence is increasing in emerging economies [8–10]. In general, HPV-positive HNSCC patients have a better prognosis than HPV-negative patients with similar tumour burden [11, 12]. More than half of these tumours are diagnosed at an advanced stage [13] and only 50% of HPV-negative patients and 66% HPV-positive patients survive up to 5 years post diagnosis (American Cancer Society, 2015). In addition, approximately 30% of HPV-positive patients develop recurrences within 2 years of initial diagnosis, whilst HPV-negative HNSCC patients show recurrences within 1 year [14]. For both tumour types when diagnosed early (before local and distant metastasis), the 5-year survival rate is 80% compared with only 15% in late diagnosis [15]. Given the clinical and biological diffrences between these two cancer types, it is therefore important to treat them separately when developing biomarkers [16].

Due to late diagnosis, the majority of HNSCC patients show either locally advanced disease and/or metastatic disease and require multimodality therapy which includes surgery, commonly followed by postoperative radiotherapy or chemo-radiotherapy [17]. In contrast, when detected early, these tumours are treated by single modality, surgery or radiotherapy alone, reducing the side effects of combining chemo-and radiotherapy. Therefore, there is an urgent need to develop early diagnostic biomarkers for the management of HNSCC patients [18].

MicroRNAs (miRNAs) are a new class of robust, small non-coding RNAs that regulate gene expression by binding to their mRNA targets [19–22]. The strong stability and altered expression levels of miRNA in biological samples (blood, saliva and urine) suggest that they could potentially be used as diagnostic, prognostic and predictive biomarkers for a number of tumour types [23, 24]. The field has moved away from using a single diagnostic biomarker to using multi-biomarker pannels [25, 26]. Multi marker panels are used to obtain an accurate overview of the tumour heterogeneity.

In a pilot study, we have previously demonstrated that three of the miRNAs from the nine panel can discriminate a healthy control group from a HNSCC patient group [27]. However, our previous data was generated using a small subet of samples, where the control group did not include smokers and ex-smokers and the patient group did not take into consideration the HPV status as well as the majority of the HNSCC patients were from an advanced stage of cancer. In contrast, the current study addresses these limitations and we have also included an additional four miRNAs, which have been preselected as these are upregulated in tumours tissue *vs* normal tissue (data obtained from The Cancer Genome Atlas) [28].

We hypothesised that the salivary miRNA panel can discriminate healthy controls from HPV-negative and HPV-positive patients as well as to differentially detect HPV-negative HNSCC patients from HPV-positive patients. The aims of this study were two-fold: (i) to identify salivary miRNAs that can discriminate a healthy control group from HPV-positive and HPV-negative HNSCC patients (ii) to identify salivary miRNAs that can differentially detect HPV-positive HNSCC patients from HPV-negative HNSCC patients.

## RESULTS

### Population characteristics

The mean age of controls was 44.7 years (SD 11.4, range 19–79 years), HPV-positive HNSCC patients was 59.7 years (SD 11.4, range 37–87 years) and that of HPV-negative HNSCC patients was 64.3 years (SD 10.38, range 42–91 years). The two genders were equally represented in the control group but in the patient group, there were more men than women. Additionally, HNSCC patients were approximately twice as likely to be smokers or having quit within the past 12 month (61.4%) compared to controls (33.6%). Only 16.8% of HNSCC patients have never been smokers compared to 62.8% of controls. Most HPV-negative HNSCC patients were current smokers (85.1%) and the minority of them were former smokers (6.4%) while within the HPV-positive HNSCC patients, 40.7% were current smokers and 35.2% former smokers. The majority of controls and HNSCC patients were Caucasians, 72.8% and 99%, respectively.

The clinical and pathological characteristics of HNSCC patients are shown in Table 1. The cancer sites were mostly of oropharyngeal and oral cavity (56.4% and 24.8% respectively), laryngeal and hypopharyngeal were 10.9% and 1% respectively; and 6.9% from other anatomical sites in the head and neck area. HPV-negative HNSCC patients had cancers within the oral cavity (42.6%) whereas the majority of HPV-positive HNSCC patients had cancers within the oropharynx (75.9%). HPV-positive HNSCC patients were mostly diagnosed (83.3%) with stages III and IV tumours, whilst 46.8% of HPV-negative patients were diagnosed with stages III and IV tumours. This is primarily due to the higher frequency of patients with nodal neck disease that is commonly seen in HPV-positive HNSCC [29].

**Table 1:** Participants demographic characteristics

	Controls (n = 113)	HPV-negative HNSCC patients (n = 47)	HPV-positive HNSCC patients (n = 54)
**Gender**			
Male	59 (52.2)	34 (72.3)	49 (90.7)
Female	54 (47.8)	13 (27.7)	5 (9.3)
**Age (years)**			
<50	73 (64.6)	5 (10.6)	10 (18.5)
50 - 60	36 (31.9)	11 (23.4)	16 (29.6)
>60	4 (3.5)	31 (65.9)	28 (51.9)
**Race and ethnicity**			
Caucasian	82 (72.6)	46 (97.9)	54 (100)
Asian	13 (11.5)	1 (2.1)	0 (0)
Other	18 (15.9)	0 (0)	0 (0)
**Smoking status**			
Smokers	38 (33.6)	40 (85.1)	22 (40.7)
*<10*	9 (7.9)	6 (12.8)	3 (5.6)
*10-20*	21 (18.6)	19 (40.4)	8 (14.8)
*>20*	8 (7.1)	15 (31.9)	11 (20.4)
Ex-smoker	4 (3.5)	3 (6.4)	19 (35.2)
Non-smoker	71 (63.7)	4 (8.5)	13 (24.1)
**Tumour characteristics**			
AJCC Stage I		6 (12.8)	3 (5.6)
Stage II		9 (19.1)	6 (11.1)
Stage III		10 (21.3)	6 (11.1)
Stage IVa		20 (42.6)	37 (68.5)
Stage IVb		1 (2.1)	2 (3.7)
Stage IVc		1 (2.1)	0 (0)
**Tumour anatomic sites**			
Oral cavity		20 (42.6)	5 (9.3)
Oropharynx		16 (34.0)	41 (75.9)
Larynx		9 (19.1)	2 (3.7)
Hypopharynx		1 (2.1)	0 (0)
Neck		1 (2.1)	6 (11.1)
**Differentiation status**			
Well differentiated		9 (19.1)	2 (3.7)
Well to moderately differentiated		2 (4.3)	1 (1.9)
Moderately differentiated		16 (34.0)	12 (22.2)
Moderately to poorly differentiated		5 (10.6)	7 (13.0)
Poorly differentiated		6 (12.8)	13 (24.1)
N/A		9 (19.1)	19 (35.2)
**Keratinisation status**			
keratinising		13 (27.7)	4 (7.4)
non-keratinising		34 (72.3)	45 (83.3)
others		0 (0)	5 (9.3)

### miRNA yields from small volumes of saliva samples

We have previously described a robust and a reproducible method to isolate high yields of miRNA from both fresh and archived saliva samples based on QIAzol (Qiagen, Valencia, CA, USA) extraction followed by solid phase enrichment of miRNAs on silica, column [27]. The amount of miRNAs isolated in 200 μL of saliva collected from healthy controls was 0.123 to 6.01 μg and from HNSCC patients was 0.21 to 19.3 μg yields.

### Salivary miRNA expression levels can discriminate a control group from a HPV-negative and a HPV-positive HNSCC patient group

**Figure 1:**
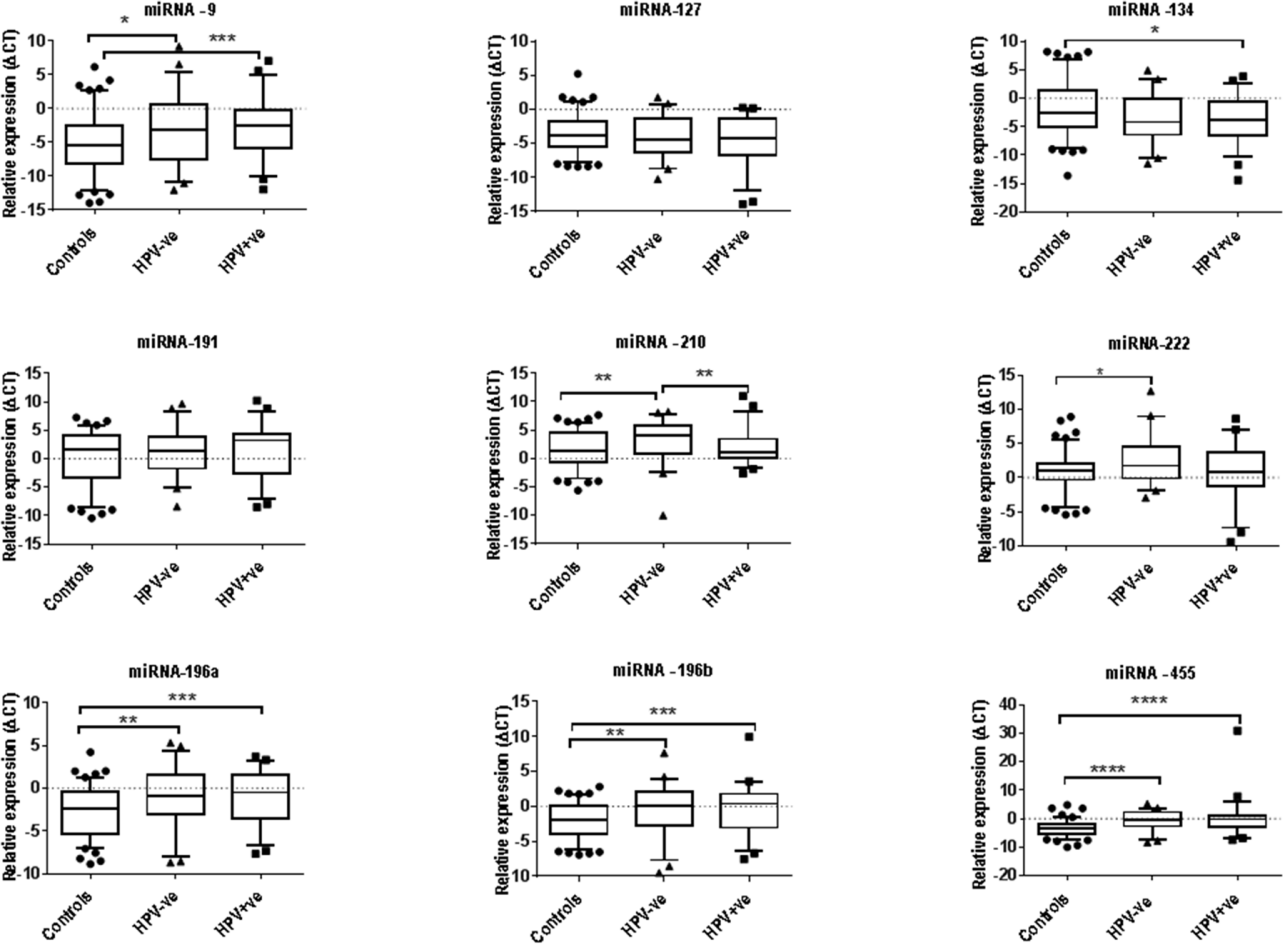
The expression levels of nine miRNA in saliva collected from healthy control (n=113) as well as HPV-negative (n=47) and HPV-positive (n=54) by RT-qPCR SNORD-96A was used as a normerliser. Statistically significant differences were determined using Mann Whitney U Test. Significant differences are denoted as ^*^P<0.05; ^**^P<0.01; ^***^P<0.001 and ^****^P<0.0001.

We have performed both univariate and multivariate statistical analysis to determine the best miRNA that can detect HPV-positive and HPV-negative HNSCC patients. Univariate analysis of individual miRNA expression levels demonstrated that miRNA-9, -196a, -196b, -210, -222, and -455 were significantly upregulated in the saliva from HPV-negative HNSCC patients compared with saliva from healthy controls; whilst the expression levels of miRNA-9, -134, -196a, -196b, -210 and -455 were significantly upregulated in the saliva from HPV-positive HNSCC patients compared with healthy controls (Figure 1).

**Figure 2:**
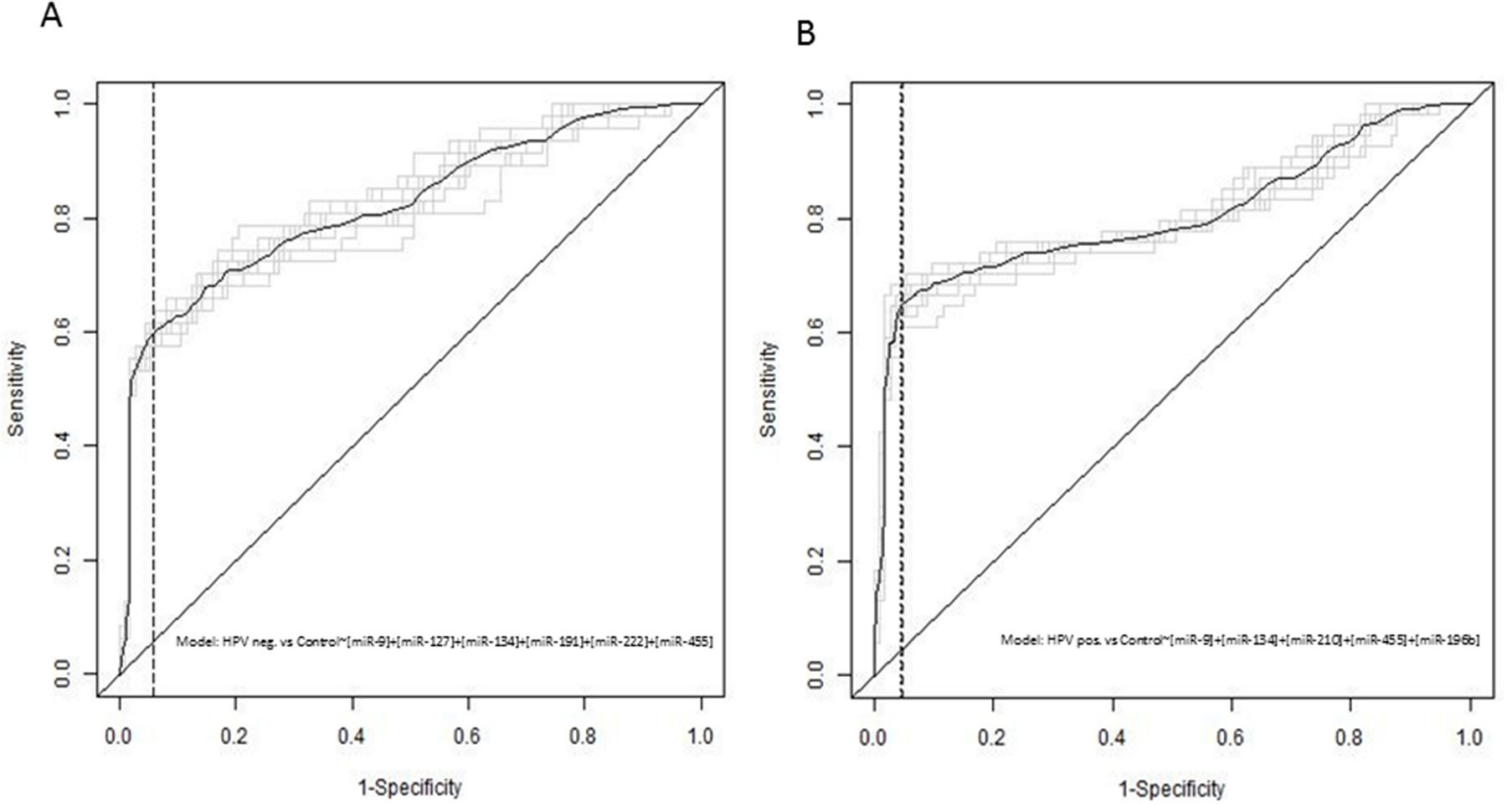
The receiver operator characteristics curve analysis from the independent validation study using saliva **(A)** HPV-negative HNSCC patients vs controls **(B)** HPV-positive HNSCC patients vs controls.

We have identified a combination of salivary miRNA-9, -127, -134, -191, -222 and -455 as the most parsimonious set of candidates for identifying HPV-negative patients from healthy controls using multiple logistic regression analysis. This resulted in 60% sensitivity and 94% specificity. The combination of miRNA-9,-134, -210, and -455 and -196b was found to be the most appropriate for identifying HPV-positive patients from healthy controls. This resulted in a sensitivity of 65% and a specificity of 95% (see Table 2 and Figure 2).

**Table 2:** Discrimination of healthy controls from HPV-negative HNSCC patients and -positive HNSCC patients using the combined miRNA panels

	Control vs HPV-patients	Control vs HPV +patients
**Probability threshold**	0.45	0.41
**Sensitivity**	0.60	0.65
**Specificity**	0.94	0.95
**Negative predictive value**	0.85	0.85
**Positive predictive value**	0.82	0.87
**AUC**	0.82	0.80

### Salivary miRNA expression levels can discriminate HPV-positive HNSCC patients from HPV-negative HNSCC patients

Using a Mann Whitney U test, the expression level of miRNA-210 was elevated in saliva collected from HPV-negative HNSCC patients compared with HPV-positive HNSCC patients. Using multiple logistic regression analysis pipeline, we have identified the combination of miRNA-191, -196b, -210, and -222 to be the most parsimonious miRNA set for discriminating HPV-negative patients from HPV-positive patients.

### Salivary miRNA expression levels could detect eraly stage HPV-negative and HPV-positive HNSCC pateints from a control group

**Figure 3:**
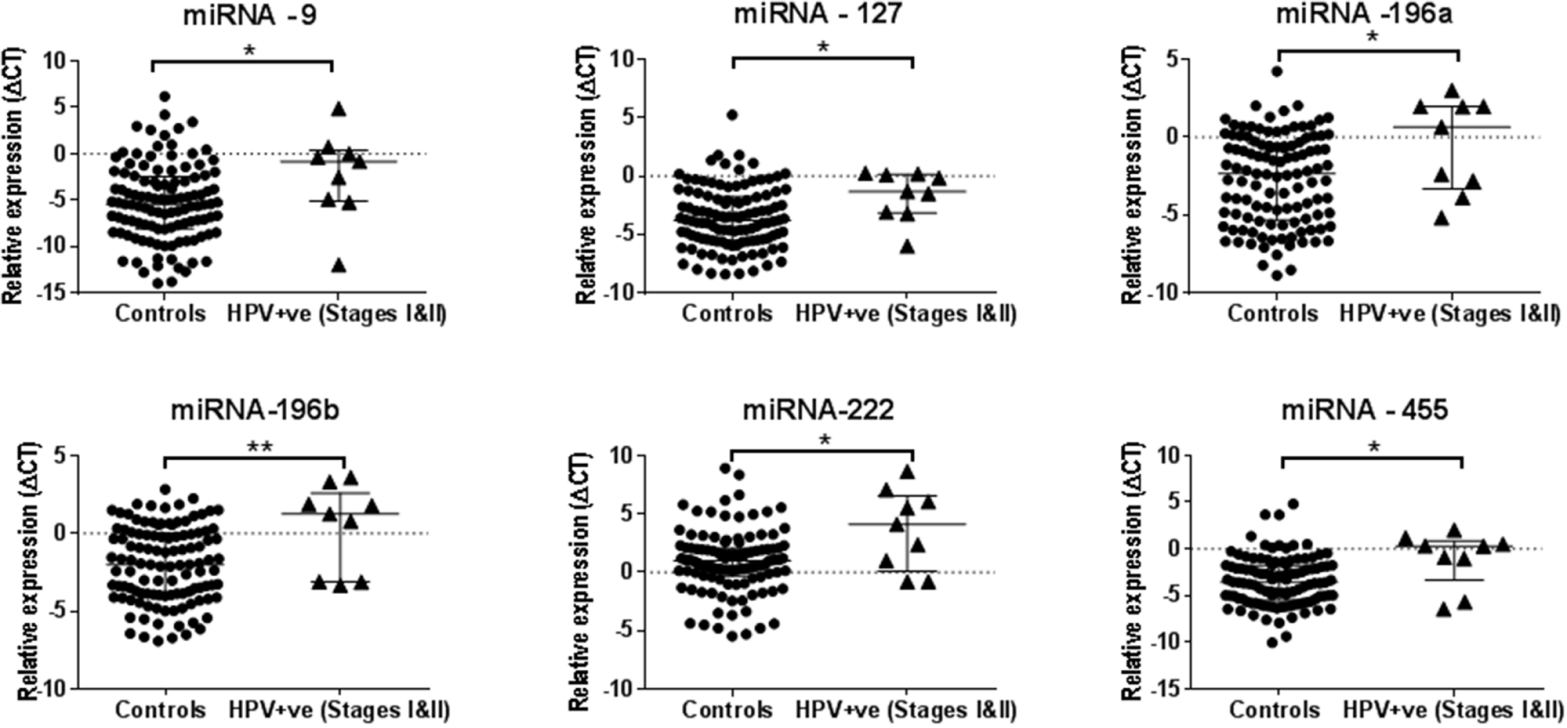
A set of six salivary miRNAs that can discriminate the early stage (Stages I&II) of HPV-positive HNSCC patients from the healthy control smoker and non-smoker group ^*^P<0.05 and ^**^P<0.01.

**Figure 4:**
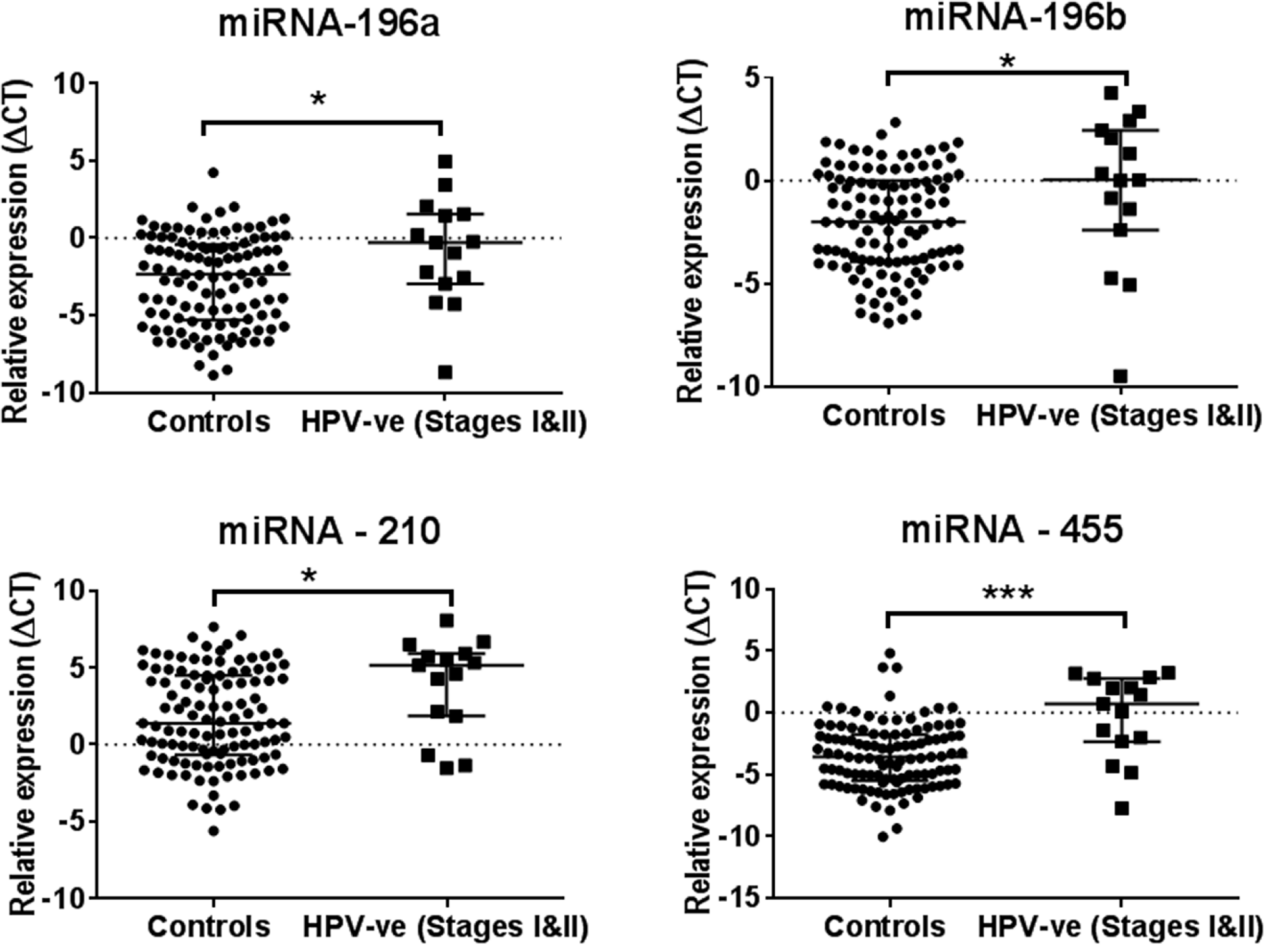
A set of four salivary miRNAs that can be used in discriminating healthy controls from the early stage (Stages I&II) of HPV-negative HNSCC patients Significant differences are ^*^P<0.05 and ^***^P<0.001.

The miRNA expression levels of -196a, -196b, -210 and -455 were found to be elevated in the saliva collected from early clinical stages (stages I and II) HPV-negative HNSCC patients compared with the saliva from healthy controls using the Mann Whitney U test. Whilst the upregulation of miRNA-9, -127, -196a, -196b, -222 and -455 were found in the saliva collected from early stage (stages I and II) HPV-positive HNSCC patients compared with healthy controls (see Figure 3 and 4).

## DISCUSSION

Despite advanced locoregional control and reduced treatment related morbidity, the five-year survival rates for HPV-positive and HPV-negative HNSCC patients have not improved [30]. Diagnostic biomarkers are urgently needed to identify these patients earlier to improve survival rates and minimise the treatment toxicity. The rationale for improving prognosis and de-escalating therapy for disease diagnosed early is not only self-evident, it is applicable to virtually every cancer, and there is overwhelming literature evidence of better prognosis with earlier diagnosis in these patients [31, 32]. In this study, a multivariable logistic regression analysis with cross validation was used to identify the most robust salivary miRNAs that can discriminate a control group from HPV-negative and HPV-positive HNSCC patients as well as to differentially diagnose HPV-negative and HPV-positive patients. In this study, we have used a multimarker approach to minimise tumour heterogeneity and biological variability.

One of the major challenges in cancer research biomarker studies is to identify stable biomolecules which can be routinely measured in easily accessible samples [33]. Robustness and reproducibility are key requisites prior to clinical implementation of biomarkers. To increase the robustness of the identified miRNA panel, we have used a pipeline consisting of multiple logistic regression with a ROC curve analysis and a 10-fold cross-validation. We have used this statistical analysis method to increase the likelihood of clinical implementation of this miRNA panel, when it is applied to an independent cohort of patients and controls. This then reduces the risk of obtaining overly optimistic data and hence increases the likelihood of clinical translation.

Previous studies have shown that miRNA expression signatures are promising biomarkers for the diagnosis, prognosis and prediction of human cancers [34–39]. We have found elevated levels of miRNA-9 in saliva collected from HNSCC patients compared with saliva from controls, paralleling tumour tissue data for HNSCC [27, 28, 40–42]. Similarily, the overexpression of miRNA-9 has been previously observed in many other cancer types including osteosarcoma, correlated significantly with poor prognosis as a result of tumor metastasis [43].

Salivary miRNA-134 expression levels showed concordance to the tumour expression levels [28]; the upregulation of miRNA-134 in tongue tumour tissues has shown to be associated with an increase in nodal metastasis as well as mortality in HNSCC patients mediated via the downregulation of *WWOX* gene [44]. In line with our salivary miRNA- 196a data, previous studies have also demonstrated an upregulation of miRNA-196a in HNSCC tissues when compared to normal tissues [45–47], with miRNA-196 precursor shown to be predictive of overall survival and disease-free survival in HPV-positive oropharyngeal cancer patients [48]. Similarly, miRNA-455 levels were elevated in saliva from HPV-positive and HPV-negative patients, supported by other studies demonstrating an elavted expression levels in tumours from uvula, larynx, floor of mouth and tongue of HNSCC patients [28, 39, 49, 50].

Current diagnosis is usually made after clinical presentation of symptoms, and involves biopsy to confirm diagnosis, HPV status by p16^INK4A^ immunostaining, and histological classification [9, 10]. These diagnostic methods are applied to advanced stage tumours with significant clinical presentation of symptoms. The treatment and clinical management also differs between HPV-negative and HPV-positive HNSCC patients. Therefore, it becomes important to discriminate between these patient groups to tailor therapy. We found that the expression levels of salivary miRNA-210 can differentiate between HPV-positive and HPV-negative HNSCC patients. In addition, infection of HPV-16/18 in organotypic raft cultures of vaginal keratinocytes increased the expression of miRNA-210 [51]. Moreover, it has been also reported that the expression levels of miRNA-127-3p is decreased in HPV-positive patients tumour tissues compared with HPV-negative patints tumour tissues [52]. Regardless of HPV status, we found that miRNA-196a, -196b and -455 were significantly overexpressed in saliva from HNSCC patients from early stages of tumour development (stages I and II). A previous study has shown that miRNA-196a plays an oncogenic role in HNSCC through deregulation of cell proliferation [47]. Similarly, another study has shown that elevated levels of miRNA-455-5p was associated with poor prognosis [53]. Albeit, our data has been generated in a small cohort of patients from stages I and II, it implies that these miRNA may have prognostic value as biomarkers for early detection of HNSCC. Therefore, a further study should be conducted to verify the predictive potential of the above markers.

One of the limitations in the current study is the low sensitivity (∼60%), in contrast to a high specificity of 90%, indicating that this panel is better suited as a screening tool [54]. In addition, age as a continouus variable may affect this panel. Current literature dipicts that miRNAs are the key regulators of aging and has shown a higher expression levels in the tissues collected from older people [55]. Intriguingly, the salivary miRNAs investigated in this study didn’t show any significant changes as a result of aging (data not shown). A lager cohort with the addition of other parameters (e.g. new miRNA or other clinical variables) is warranted for a further investigation.

In summary, we have identified two miRNA panels that can discriminate healthy controls from HPV-negative (sensitivity =60%; specificity =94%) and -positive HNSCC patients (sensitivity =65%, specificity =95%). Remarkably, the salivary miRNA data strongly correlated with published TCGA data using tumour tissues obtained from HNSCC patients (international studies). Therefore, we are confident that the miRNA expression changes in saliva can be used as a proxy to determine tumour progression. Our approach of using multiple logistic regression coupled to ROC analysis with cross-fold validation has allowed us to identify salivary miRNAs with minimal biological variations in a control and a patient cohort. This opens up new avenues for exploring the utility of salivary miRNAs as either diagnostic, prognostic indicators or to determine the response to treatment. A future multi-centre confirmatory study is warranted to test the diagnostic performance of these salivary miRNA prior to clinical implementation.

## MATERIALS AND METHODS

### Participants

Unstimulated whole mouth saliva samples were collected from healthy individuals, including smokers and non-smokers (n=113) with no previous history of any malignancies to the head and neck areas as well as from HPV-positive (n=54) and HPV-negative (n=47) HNSCC patients at various tumour nodal metastasis (TNM) clinical stages (stages II-IV) of cancer who presented to the Head and Neck Clinic at the Princess Alexandra Hospital, Woolloongabba, Australia. Patients with early stages of tumours (stages I-II) in general do not show either local and/or distant metastasis [56]. The participants’ demographics and clinical characteristics are shown in Table 1. This study was approved by the University of Queensland Medical Ethical Institutional Board [HREC No: 2014000679 and 2014000862] and the Queensland University of technology [HREC No: 1400000617 and 1400000641] and by the Princess Alexandra Hospital Ethics Review Board [HREC Number: HREC/12/QPAH/381]. All participants gave written informed consent before sample collection.

### Saliva sample collection and processing

Whole mouth resting saliva samples were collected from volunteers while seated in an upright position. Volunteers were asked to rinse their mouths with drinking water to remove food debris. They were then asked to tilt their heads down and pool saliva in the mouth for 2-5 minutes [57, 58] before drooling into sterile 50 mL Falcon tubes (Sarstedt, Australia). Saliva samples were transported to the laboratory on dry ice.

### miRNA extraction and cDNA synthesis from saliva samples

The enrichment and isolation of miRNA from saliva samples was performed using the NucleoSpin miRNA kit (Machnery-Nagel, Germany) as previously published [27]. In brief, whole mouth saliva (200 μL) was added to 800 μL of QIAzol lysis reagent (Qiagen, Valencia, CA, USA). The samples were briefly vortexed and incubated for 5 minutes at room temperature, 200 μL of chloroform was added and vortexed vigorously and incubated for a further 5 minutes at RT. Samples were then centrifuged at 10,000 x g for 10 minutes at 4°C. An aliquot of the upper aqueous layer (800 μL) was transferred into a new micro centrifuge tube and a further 200 μL of chloroform was added and vortexed. Samples were then centrifuged again at 10,000 x g at 4°C. The upper aqueous layer containing total RNA was carefully removed (800 μL) and transferred into a new micro centrifuge Eppendorff tube and 200 μL of 100% ethanol was added. The solution containing total RNA was passed through Nucleospin column (blue ring) by centrifugation at 11,000 g for 30s at RT. The eluent was collected and 800 μL of MX buffer was added.

The sample was loaded onto a Nucleospin column (green ring) and centrifuged at 11,000 g for 30 s at RT. The columns were then washed with 600 μL of MW1 buffer and centrifuged at 11,000 g for 30 s. The wash was repeated with 700 μL followed by 250 μL of MW2 buffer and centrifuged at 11,000 for 30 s. The columns were then centrifuged at 11,000g for 2 mins to remove all traces of ethanol. The miRNA was eluted in 30 μL of RNase free Water. The quantity of the isolated miRNA was determined by a Nanodrop ND-1000 spectrophotometer (Thermo scientific Wilmington DE).

The cDNA synthesis of isolated miRNA (with an input of 250 ng) was carried out using the miScript II RT Kit with the Hispec buffer (Qiagen, Valencia, CA, USA) according to the manufacturers’ instructions. In brief, the miRNAs were polyadenylated by polymerase A and transcribed into cDNA using oligo-dT primers, both processes took place in parallel in the same tube. The oligo-dT has a 3’degenerate anchor and a universal tag sequence on the 5’end allowing amplification of mature miRNA using real-time PCR. The polyadenylation and universal tag primer ensured that genomic DNA was not amplified and so it was not necessary to perform a DNase treatment in the samples.

### Quantitative real-time RT-PCR (RT-qPCR) for nine miRNA panel

To validate the miRNA expression levels in saliva samples, the nine miRNA miScript™ primer PCR Assays (Qiagen, Valencia, CA, USA) were used. This miRNA PCR panel included: hsa-miRNA-210-5p (Cat. No. MS00003801); hsa-miRNA-222-3p (Cat. No. MS00007609); hsa-miRNA-134-3p (Cat. No. MS00031437); hsa-miRNA-127-5p (Cat. No. MS00008575); hsa-miRNA-191-5p (Cat. No. MS00003682); hsa-miRNA-9-5p (Cat. No. MS00010752); hsa-miRNA-196a-3p (Cat. No. MS00031563); hsa-miRNA-196b-3p (Cat. No. MS00031570) and hsa-miRNA-455-3p (Cat. No. MS00009744). Briefly, 3 ng cDNA was used as a template in PCR amplifications and PCRs were run in duplicate using miScript Syber green PCR master mix (Qiagen, Valencia, CA, USA). The PCR was performed using the QuantStudio™ 7 Flex Real-Time PCR System (Thermo Fisher Scientific, Rockford, ILUSA). The PCR reaction mixtures were incubated at 95°C for 15 min to activate hot start Taq DNA polymerase and this was followed by 40 cycles of 94°C for 15 s, 55°C for 30s and 70°C for 30s, monitored by melt curve analysis. The threshold value (Ct) was determined for each well and the Ct values were averaged for each gene according to the Minimum Information for Publication of Quantitative Real-Time PCR Experiments guidelines [59].

A panel of five miRNAs were also tested for suitability as normalisers for miRNAs detected in human saliva (see Supplementary Table 1a). These assays are wet-benched validated miScript™ primer PCR Assays from Qiagen and included RNU6.2 (Cat. No. MS00033740); SNORD68 (Cat. No. MS00033712); SNORD72 (Cat. No. MS00033719); SNORD95 (Cat. No. MS00033726) and SNORD96A (Cat. No. MS00033733). We found that SNORD-96A demonstrated minimal Ct changes between saliva collected and analysed from healthy controls and the patients. As such SNORD96A was used as a normaliser in all subsequent work. The average relative expression (∆Ct) of each targeted miRNA in controls and patients is listed in Supplementary Table 1b.

### Statistical analysis

The expression levels of individual miRNAs from HPV-negative and HPV-positive HNSCC patients and the healthy controls were compared using the non-parametric Mann Whitney U test, using GraphPad Prism 5 software version 5.03 (GraphPad Software Inc., USA). The clinical utility of miRNAs as biomarkers was investigated using R [60] and RWeka [61]. A logistic regression model was fitted using RWeka [61], with the patient status as the outcome variable and the miRNAs as explanatory variables. RWeka used a variable selection procedure based on the cross validation error for selecting the most parsimonious model. This model was then used in 10 runs of 10-fold cross validation using the “train” function of the “Caret” package [62]. Since each cross validation randomly creates 10 folds (splits) of the data, the 10 runs could be considered to be different replicates of the same procedure [63]. The reader is directed to the data mining book of Witten et al., (2011) for further information. For each of these 10 runs, the predictions of this model and the performance were created and evaluated using the “predict” and “performance” functions respectively of the ROCR package [63]. Several methods have been proposed for averaging the ROCs curves [64]. In this study, threshold averaging was used, since it averaged the curves at exactly the same thresholds.

## SUPPLEMENTARY MATERIALS TABLES



## Abbreviations

HNSCC: Head and neck squamous cell carcinomas
OPC: oropharyngeal cancers
HPV-16: human papillomavirus type 16
SCC: squamous cell carcinoma
OC: oral cavity cancers
TNM: tumour nodal metastasis
HREC: Human Research Ethics Committees
miRNAs: MicroRNAs
RT: Reverse transcription
cDNA: complementary DNA
qPCR: quantitative PCR
Ct: cycle threshold
ROC: receiver operating characteristic
AUC: area under the curve
