# Supplementary material for: Salivary miRNA panel to detect HPV-positive and HPV-negative head and neck cancer patients
Source: Oncotarget. 2017 Oct 10;8(59):99990–100001. doi: 10.18632/oncotarget.21725 (PMC5725146; doi:10.18632/oncotarget.21725)
Supplement: Supplementary file 1 [file oncotarget-08-99990-s001.pdf]

Salivary miRNA panel to detect HPV-positive and HPV-negative head and neck cancer patients

SUPPLEMENTARY MATERIALS

Supplementary Table 1a: The qPCR thresh-hold (Ct) values for a panel of miRNA normalisers

Group 1 (Controls)	SNORD96a	RNU6.2	SNORD68	SNORD72	SNORD95
Control 1	26.8	27.7	27.8	34.2	24.1
Control 2	26.2	30.4	29.1	33.9	24.9
Control 3	31.2	31.1	29.2	35.8	25.9
Control 4	24.9	36.9	35.1	40.0	30.3
**Average**	**27.3**	**31.5**	**30.3**	**36.0**	**26.3**
**SD**	**2.7**	**3.9**	**3.3**	**2.8**	**2.8**
**Group 2 (HNSCC patients)**					
Patient 1	31.7	36.4	37.5	Undetermined	32.6
Patient 2	25.0	36.3	36.1	Undetermined	33.0
Patient 3	23.0	Undetermined	35.0	Undetermined	32.0
Patient 4	31.5	30.1	31.0	Undetermined	27.6
Patient 5	22.1	34.4	33.3	Undetermined	29.7
**Average**	**26.7**	**35.4**	**34.6**	Undetermined	**31.0**
**SD**	**4.3**	**3.1**	**2.2**		**2.4**
**M**	0.30	0.16	0.13		0.13

Undetermined >40 Ct values. SD = standard deviation and M = a stability score.

Supplementary Table 1b: The average relative expression (ΔCt) represents the average expression level of each salivary miRNA in healthy control, HPV-negative (n=47) and HPV-positive HNSCC patients

	Controls (n=113)		HPV-ve patients (n=47)		HPV+ve patients (n=54)	
	Average ΔCt	SD	Average ΔCt	SD	Average ΔCt	SD
miRNA-9	-5.28	4.15	-3.33	5.08	-2.66	4.42
miRNA-127	-3.59	2.60	-4.09	2.98	-4.69	3.78
miRNA-134	-1.98	4.57	-3.49	4.05	-3.75	4.08
miRNA-191	0.03	4.74	1.14	3.91	1.55	4.42
miRNA-222	0.91	2.69	2.41	3.37	0.79	3.95
miRNA-210	1.70	3.04	3.16	3.54	1.75	2.66
miRNA-455	-3.46	2.63	-0.60	3.36	-0.43	5.43
miRNA-196a	-2.72	2.87	-1.20	3.46	-1.03	3.02
miRNA1-96b	-2.01	2.43	-0.62	3.54	-0.29	3.23
